# Prevention of the Musculoskeletal Complications of Hemophilia

**DOI:** 10.1155/2012/201271

**Published:** 2012-06-14

**Authors:** E. C. Rodriguez-Merchan

**Affiliations:** Department of Orthopaedic Surgery, La Paz University Hospital, Paseo de la Castellana 261, 28046 Madrid, Spain

## Abstract

Hemophilia is an inherited disorder of clotting factor deficiencies resulting in musculoskeletal bleeding, including hemarthroses, leading to musculoskeletal complications. The articular problems of hemophiliac patients begin in infancy. These include: recurrent hemarthroses, chronic synovitis, flexion deformities, hypertrophy of the growth epiphyses, damage to the articular cartilage, and hemophilic arthropathy. The most commonly affected joints are the ankle, the knee, and the elbow. Hematologic prophylactic treatment from ages 2 to 18 years could avoid the development of hemophilic arthropathy if the concentration of the patient's deficient factor is prevented from falling below 1% of normal. Hemarthroses can be prevented by the administration of clotting factor concentrates (prophylaxis). However, high costs and the need for venous access devices in younger children continue to complicate recommendations for universal prophylaxis. Prevention of joint arthropathy needs to focus on prevention of hemarthroses through prophylaxis, identifying early joint disease through the optimal use of cost-effective imaging modalities and the validation of serological markers of joint arthropathy. Screening for effects on bone health and optimal management of pain to improve quality of life are, likewise, important issues. Major hemarthrosis and chronic hemophilic synovitis should be treated aggressively to prevent hemophilic arthropathy.

## 1. Introduction

Hemophilia is an inherited disorder of clotting factor deficiencies resulting in musculoskeletal bleeding, including hemarthroses, leading to musculoskeletal complications [1]. The pathogenesis of hemophilic joint arthropathy continues to be explored and there is evidence to suggest that iron, cytokines, and neoangiogenesis can initiate synovial and early cartilage damage resulting in molecular changes and the perpetuation of a chronic inflammatory state. This joint arthropathy has long-term consequences for bone health resulting in chronic pain and quality of life issues in the individual with hemophilia.

Hemophilia has been recognized as the most severe among the inherited disorders of blood coagulation since the beginning of the first millennium [2]. Joint damage is the hallmark of the disease. Despite its frequency and severity, the pathobiology of blood-induced joint disease remains obscure. Although bleeding into the joint is the ultimate provocation, the stimulus within the blood inciting the process and the mechanisms by which bleeding into a joint results in synovial inflammation (synovitis) and cartilage, and bone destruction (arthropathy) are unknown. Clues from careful observation of patient material, supplemented with data from animal models of joint disease, provide some clues as to the pathogenesis of the process. 

**Figure 1:**
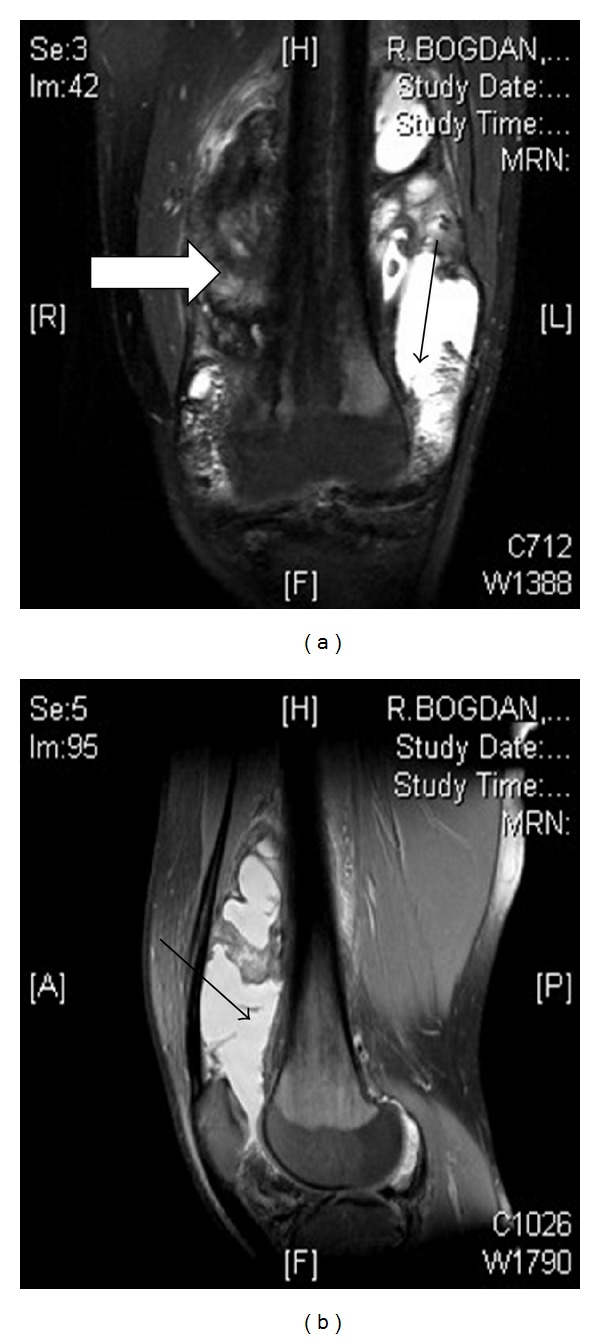
MRI of the knee joint of a 27-year-old haemophiliac. In the AP view (a) intra-articular blood can be noted in the lateral side of the joint (black arrow), while in the medial side a severe degree of synovitis can be seen (white arrow). In the lateral view of the MRI (b), the aforementioned hemarthrosis can also be noted (black arrow).

The articular problems of hemophiliac patients begin in infancy. These include recurrent hemarthroses, chronic synovitis, flexion deformities, hypertrophy of the growth epiphyses, damage to the articular cartilage and hemophilic arthropathy. The most commonly affected joints are the ankle, the knee, the elbow, and the hip. The hemarthroses tend to persist despite the reabsorptive properties of the synovium which eventually becomes hypertrophic and more prone to injury, leading to a vicious circle of bleeding, synovitis, and more bleeding (Figure 1). The pain causes flexion deformities in affected joints, first correctable, but later becoming fixed. The hyperemic reaction to the hemarthrosis produces hypertrophy of the growth epiphyses. This is often asymmetrical, producing a valgus deformity at the involved joint. Both factors lead to damage to the articular cartilage, which evolves into the destruction of the joint, known as hemophilic arthropathy [3, 4]. The purpose of this paper is to revise the current prevention of the musculoskeletal complications of hemophilia.

## 2. Musculoskeletal Complications of Hemophilia

### 2.1. Hemarthroses

The correct management of hemophilic hemarthrosis should include prompt diagnosis, adequate hematological treatment, joint aspiration, physiotherapy and avoidance of rebleeding. Patients with hemarthrosis commonly feel a tingling sensation—the “aura”—before the episode of intraarticular bleeding. The joint becomes warm, swollen, very painful and with an antialgic position in flexion. Clinical diagnosis should be confirmed by means of MRI and/or ultrasonography (US). Radiographs should also be performed, looking for any evidence of radiological involvement. Until recently hemarthroses have been treated by means of intravenous injection of 20–30 U/kg body weight of the deficient coagulation factor under hematological control, short-term rest and immobilization in the antialgic position by means of bandages, plaster splints, bed rest, and analgesics. Only 20% of the countries around the world have sufficient economical power to give their hemophilia population on-demand substitutive therapy. This consists of the intravenous injection of 20–30 units of Factor VIII/kg body weight when a bleed occurs, until the symptoms of an acute hemarthrosis abate [5]. 

Joint aspiration of hemophilic hemarthrosis remains a controversial issue. Until recently it was considered an extremely dangerous procedure to perform, with a high risk of re-bleeding and infection (septic arthritis). Today, I believe in the efficacy of early joint aspiration. However, the technique must be performed under hematological control and aseptic conditions. The procedure must be repeated many times in the patient's life, starting at a very short age, and it carries some difficulties; therefore, psychological and familiar support is paramount. It is important that the child trusts the orthopedic surgeon carrying out the joint aspiration and some form of local anesthesia should be used in order to minimize pain. Following the procedure, immobilization is recommended for 3–5 days by means of a compressive bandage. Later on, the patient should start a supervised period of physiotherapy as rehabilitation is paramount to halt the development of synovitis. The duration of physiotherapy will depend on the time required to regain full range of movement and muscular strength. Re-bleedings during the recovery period should be avoided as much as possible. Patients must be seen every 3 months at the outpatient clinic for close and careful assessment [6].

### 2.2. Chronic Hemophilic Synovitis

The objectives of treatment are to stop the hemarthroses or to control them quickly and to avoid secondary synovitis. Once synovitis has developed, which is inevitable, the aim is to treat it as early and aggressively as possible. Confirmation of the diagnosis is important and can be achieved by ultrasound or MRI. The former is especially useful for the knee, but MRI gives greater precision for the elbow and the ankle. Sometimes standard conservative measures, such as factor replacement and physiotherapy, do not break the vicious cycle of hemarthrosis-synovitis-hemarthrosis. Under these circumstances synovectomy—either chemical, radioactive, or surgical (open or arthroscopic), can reduce the bleeding tendency and so delay the onset of hemophilic arthropathy. 

Currently we perform ablation of the synovium as soon as synovitis is diagnosed. There are both conservative (RS) and operative methods (synovectomy). The drugs most commonly used in RS are radioactive isotopes such as Yttrium-90 and Phosphorus-32 [7, 8]. In my experience, synovectomy (by any method) reduces the tendency to bleeding episodes, but does not halt the deterioration of joints. RS should be the first choice for patients with persistent synovitis of the joints. If two to three consecutive RSs at 6 month intervals fail to halt synovitis, an arthroscopic synovectomy should be considered as an alternative to the treatment of chronic hemophilic synovitis. 

Radiosynovectomy (RS) affords effective treatment of chronic hemophilic synovitis. RS is effective in all patient groups, independently of the presence of circulating inhibitor antibody, the type of joint involved, the degree of synovial membrane hypertrophy, and the presence of arthropathy [9]. 

RS is a safe, simple, and effective method for the treatment of chronic haemophilic synovitis. RS with Yttium-90 and rhenium-186 has been shown to decrease the number of bleeding episodes, joint pain, the size of the synovium (clinically and radiologically), muscle strength (MS), ROM, and the WFH clinical score [10]. Nonetheless, RS did not succeed in improving the radiological score. The parameters mentioned improved independently for each one of the intra-articular radioisotope injections performed. Categorizing the different variables attending to the degree of improvement achieved after RS showed that hemarthrosis and pain were the variables undergoing the greatest improvement, with a decrease in bleeding and on the WFH pain scale of around 70%. Synovial hypertrophy, as assessed clinically or radiologically, also showed a clear improvement (between 30 and 40%). The WFH clinical scale improved by around 20%. MS also improved with an increase of around 10%. ROM experienced a slight yet nonsignificant improvement both in flexion and in extension. The WFH radiological score showed no improvement. RS with Yttrium-90 in knees and Rhenium-186 in elbows and ankles is effective in hemophilic patients with chronic synovitis, regardless of the type of joint involved and the degree of synovitis present. Nevertheless, a study also showed that the knee joint and the more severe cases of synovitis require a higher number of RS injections [11]. 

### 2.3. Articular Deformities, Subchondral Cysts, and Osteophytes

As already stated, an uncontrolled hemarthrosis and synovitis will lead to flexion deformity, sometimes with valgus deviation caused by asymmetrical growth of the epiphyses, Perthes' disease, osteophytes, and subchondral cysts. In infants, adolescents and young adults with flexion deformity, realignment osteotomies may be indicated to prevent development of severe arthropathy [12, 13]. Flexion deformities should be managed by early physiotherapy, traction, orthoses, tendon and capsular release, or extension osteotomy. Flexion deformity of the knee requires early physiotherapy and the use of progressive traction in extension and appropriate orthoses. 

At the hip, a form of Perthes' disease may develop related to recurrent intra-articular bleeding. The initial treatment should be by means of an ambulation-abduction bracing. Because of intraosseous hemorrhages, some hemophiliac patients develop large symptomatic juxta-articular cysts, especially in the proximal tibia. These may threaten the integrity of the articular cartilage and require open curettage and grafting with the use of fibrin sealant [14]. At the ankle some patients develop a large anterior osteophyte; surgical excision of this can give relief of symptoms [15].

### 2.4. Advanced Hemophilic Arthropathy 

**Figure 2:**
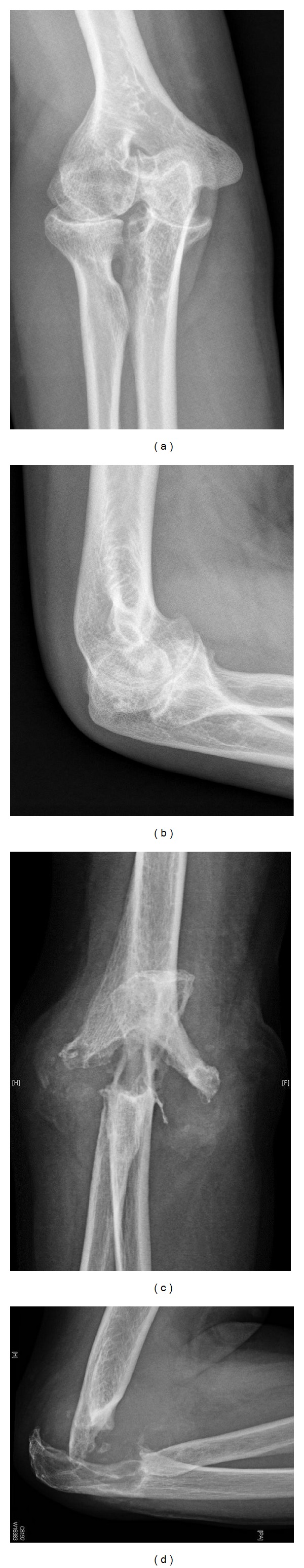
Hemophilic arthropathy of the elbow. At the age of 29 a severe degree of arthropathy was already seen in the AP radiograph (a) and in the lateral view (b). Forty years later the joint was fully destroyed both in the AP view (c) as in the lateral radiograph (d).

**Figure 3:**
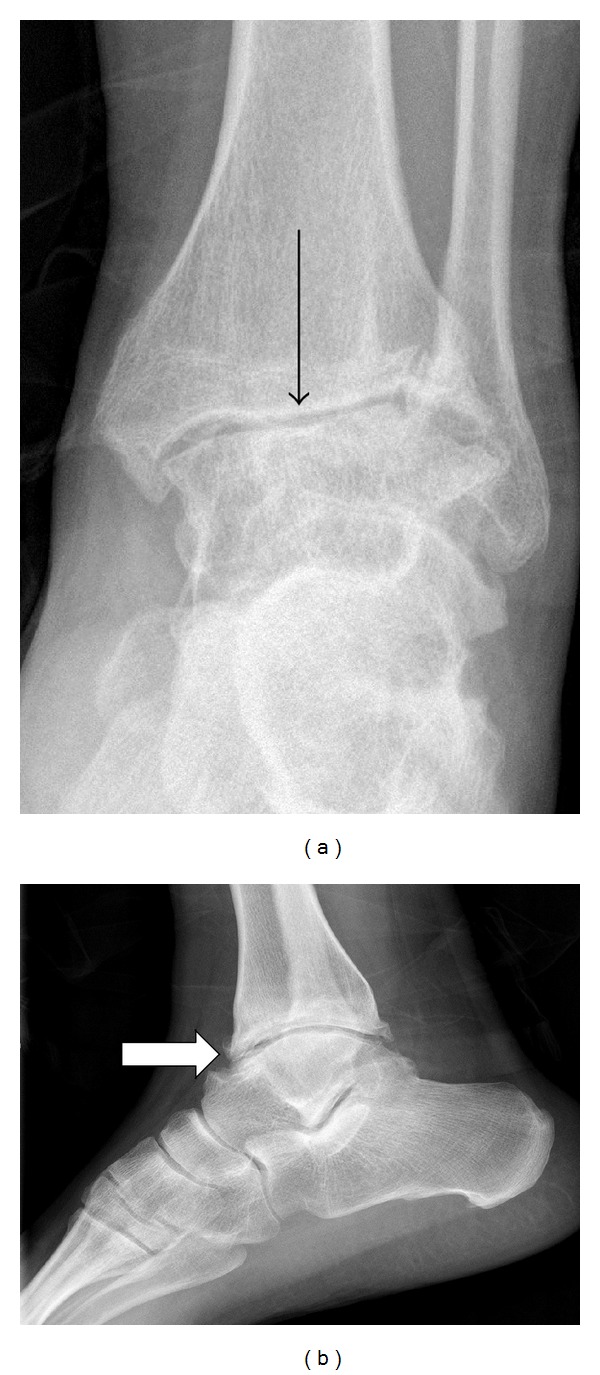
Hemophilic arthropathy of the ankle (black arrow) in the AP (a) and lateral (white arrow) radiographs (b).

**Figure 4:**
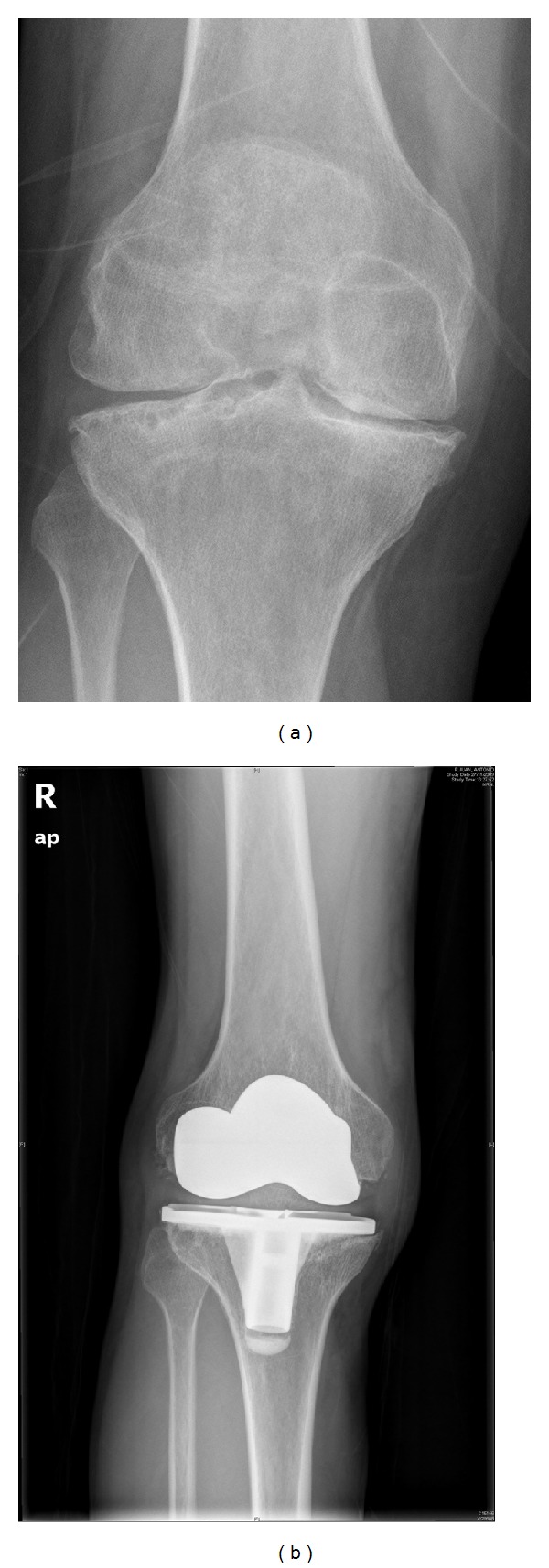
Severe arthropathy of the knee joint (a) that required a total knee arthroplasty (b), with a satisfactory result.

Between the second and fourth decades, many hemophiliacs develop severe articular destruction (Figures 2 and 3). At this stage, possible treatments include resection of the radial head, total hip arthroplasty, open knee debridement and total knee arthroplasty (Figure 4), and ankle arthrodesis [5]. In polyarthritic conditions, the repair of a single joint may not improve functional ability, and the aim should be to create a functional limb. Horoszowski et al. reported the use of multiple joint procedures on hemophiliac patients in a single operative session [16]. This succeeded in achieving a functional limb. The complication rate was lower than expected and the rehabilitation period was relatively short. 

At the mature elbow, the resection of a hypertrophic radial head usually reduces the incidence of recurrent hemorrhages and improves the range of pronation-supination of the affected joint. For the hip the best solution is a total hip arthroplasty [5, 17, 18]. 

### 2.5. Muscle Hematomas and Pseudotumors

**Figure 5:**
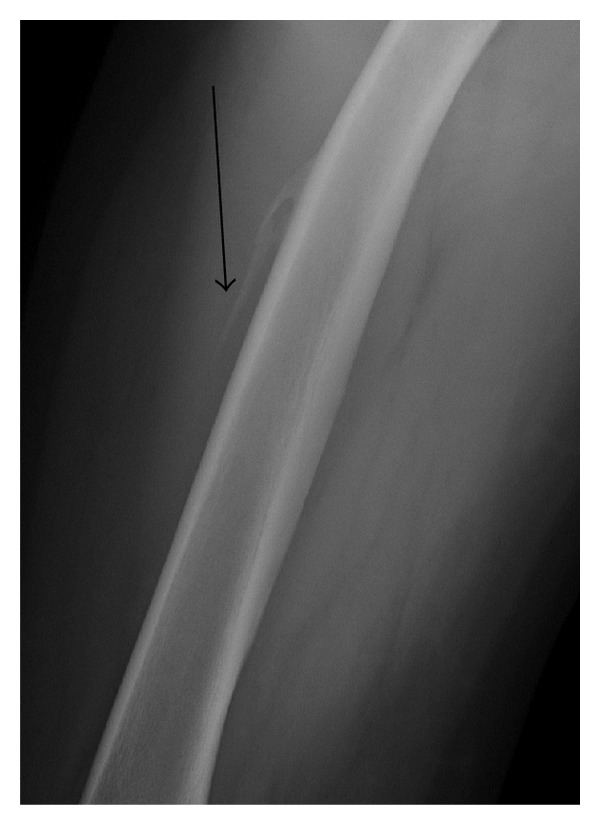
Subperiosteal hematoma (black arrow) of the thigh in a 27-year-old person with hemophilia.

Bleeds within the muscles are very often associated with direct trauma and the pathology becomes quite evident due to the swelling, pain, local warmth, and bruising that typically appear in the overlying skin (Figure 5). The vast majority of these muscle bleeds resolve spontaneously, leaving no functional loss. It is, however, necessary to examine the patient carefully to ensure that there is no complications (compartment syndromes and pseudotumours).

Pseudotumor is a serious, but very rare, complication. A progressive cystic swelling involving muscle is produced by recurrent bleeding and there is usually radiological evidence of bone involvement. Most are in adults near the large bones of the proximal skeleton. A few develop distal to the wrist and ankle in younger patients before skeletal maturity. Untreated proximal pseudotumors will destroy soft tissues, erode bone and produce vascular or neurological lesions. 

## 3. Prevention of the Musculo-Skeletal Complications of Hemophilia

### 3.1. Patients without Inhibitors

Recurrent haemarthroses in patients with severe and moderate hemophilia can result in the development of one or more target joints and subsequent degenerative joint disease [19]. This debilitating process is characterized by physical and physiological changes in articular cartilage, synovium, and bone. Efforts to prevent or limit arthropathy include the use of prophylactic factor infusion regimens, surgical joint intervention, or both.

Prevention of arthropathy is a major goal of hemophilia treatment. While studies in adults have demonstrated an impact of prophylaxis on the incidence of joint bleeds and patients' well-being in terms of improved quality of life (QoL), it is unclear whether or not prophylaxis influences the outcome and perception of well-of children with hemophilia [20]. Gringeri et al. compared the efficacy of prophylaxis with episodic therapy in preventing hemarthroses and image-proven joint damage in children with severe hemophilia A (factor VIII <1%) over a 10-year time period. Forty-five children with severe hemophilia A, aged 1–7 years (median 4), with negative clinical-radiologic joint score at entry and at least one bleed during the previous 6 months, were consecutively randomized to prophylaxis with recombinant factor VIII (25 IU kg(−1) 3 × week) or episodic therapy with ≥25 IU kg(−1) every 12–24 h until complete clinical bleeding resolution. Safety, feasibility, direct costs, and QoL were also evaluated. Twenty-one children were assigned to prophylaxis, 19 to episodic treatment. Children on prophylaxis had fewer hemarthroses than children on episodic therapy: 0.20 versus 0.52 events per patient per month. Plain-film radiology showed signs of arthropathy in six patients on prophylaxis (29%) versus 14 on episodic treatment (74%). Prophylaxis was more effective when started early (≤36 months), with patients having fewer joint bleeds (0.12 joint bleeds per patient per month) and no radiologic signs of arthropathy. This randomized trial confirmed the efficacy of prophylaxis in preventing bleeds and arthropathy in children with hemophilia, particularly when it is initiated early in life.

It has been shown that patients with severe hemophilia treated on demand are not as physically active as their healthy peers and often have a sedentary lifestyle that contributes to chronic joint disease [21]. The use of prophylaxis provides opportunities for participation in physical activities with fewer bleeding episodes. The objective of the study was to describe the type, intensity, and duration of physical activity among adult patients with severe hemophilia and to find out whether a joint function dependency exists. Patients with severe hemophilia, divided into two groups (group A: patients who started prophylaxis at the age of ≤3 years and group B: patients who started prophylaxis at the age of >3 years), and 190 controls were included. Physical activity was assessed using the self-report Modifiable Activity Questionnaire. Time involved and intensity of all aspects of physical activity for group A were almost similar to their healthy peers. Group B had significantly lower vigorous, leisure, and total physical activities than group A and their healthy peers. Positive significant correlations were found between leisure and total physical activities and joint score in group A, whereas in group B, there was negative significant correlation between only nonweight-bearing activity and joint score. The early start of long-term, primary prophylaxis has been successful in reducing frequency of bleeds and thereby preventing or delaying subsequent chronic joint disease and enables the patients to lead a physically normal life also during adulthood when patients with hemophilia treated on demand are expected to have substantial joint disease impacting their physical activity.

The Spanish Epidemiological Study in Hemophilia carried out in 2006 enrolled 2400 patients (2081–86.7% with haemophilia A and 319–13.3% with haemophilia B [22]); 465 of them (19.4%) were on prophylaxis. These rates were higher in patients with severe hemophilia (45.4%) and severe paediatric cases (72.5%). On the basis of information recorded in this study, they analysed the current situation of prophylaxis therapy administered to patients with hemophilia A in Spain, as well as their orthopaedic status. Prophylaxis was used in 399 (19.2%) patients with hemophilia A; such prophylaxis was primary in 20.3% and secondary in 75.9% of cases. Among severe hemophilia A patients, 313 (45.9%) were on prophylaxis (22.3% on primary prophylaxis and 74.7% on secondary prophylaxis). Taking into account the patients' age, 34.7% of severe hemophilia A adults were on prophylaxis (6% primary prophylaxis and 92.1% secondary prophylaxis), whereas 71.5% of severe hemophilia A pediatric patients (40.5% primary prophylaxis and 55.4% secondary prophylaxis) received this kind of treatment. Established hemophilic arthropathy was detected in 142 from 313 severe hemophilia A patients (45.3%) on prophylaxis, but only in 2.9% of patients under primary prophylaxis versus 59% of patients receiving secondary prophylaxis. There was no established hemophilic arthropathy in adult severe hemophilia A patient on primary prophylaxis, whereas 70.4% on secondary prophylaxis had joint damage. Among pediatric severe hemophilia A patients, established hemophilic arthropathy was detected in 3.3% under primary prophylaxis and 37.8% under secondary prophylaxis. Lucia et al. suggested that an early initiation of prophylaxis avoids established hemophilic arthropathy in the long term in patients with severe hemophilia A. They emphasized the early onset of prophylaxis regimens.

### 3.2. Patients with Inhibitors

Neutralizing inhibitors develop in 20–30% of patients with severe factor VIII (FVIII) deficiency. It is well established that Blacks have a higher prevalence of inhibitors than Whites [23]. The prevalence of high-titre inhibitors in the Hispanic participants is 24.5% compared to 16.4% for White non-Hispanic patients. 

Patients with severe hemophilia A and factor VIII inhibitors are at increased risk for serious bleeding complications and progression to end-stage joint disease. Effective strategies to prevent bleeding in such patients have not yet been established. Leissinger et al. [23] enrolled patients with hemophilia A who were older than 2 years of age, had high-titer inhibitors, and used concentrates known as bypassing agents for bleeding in a prospective, randomized, crossover study comparing 6 months of anti-inhibitor coagulant complex (AICC), infused prophylactically at a target dose of 85 U per kilogram of body weight (±15%) on 3 nonconsecutive days per week, with 6 months of on-demand therapy (AICC at a target dose of 85 U per kilogram [±15%] used for bleeding episodes). The two treatment periods were separated by a 3-month washout period, during which patients received on-demand therapy for bleeding. The primary outcome was the number of bleeding episodes during each 6-month treatment period. Thirty-four patients underwent randomization; 26 patients completed both treatment periods and could be evaluated per protocol for the efficacy analysis. As compared with on-demand therapy, prophylaxis was associated with a 62% reduction in all bleeding episodes, a 61% reduction in hemarthroses, and a 72% reduction in target-joint bleeding (≥3 hemarthroses in a single joint during a 6-month treatment period). Thirty-three randomly assigned patients received at least one infusion of the study drug and were evaluated for safety. One patient had an allergic reaction to the study drug. AICC prophylaxis at the dosage evaluated significantly and safely decreased the frequency of joint and other bleeding events in patients with severe hemophilia A and factor VIII inhibitors.

### 3.3. Pharmacoeconomics of Prophylaxis

Health economic evaluations provide valuable information for healthcare providers, facilitating the treatment decision-making process in a climate where demand for healthcare exceeds the supply [24]. Although an uncommon disease, hemophilia is a life-long condition that places a considerable burden on patients, healthcare systems, and society. This burden is particularly large for patients with hemophilia with inhibitors, who can develop serious bleeding complications unresponsive to standard factor replacement therapies. Hence, bleeding episodes in these patients are treated with bypassing agents such as recombinant activated FVII (rFVIIa) and plasma-derived activated prothrombin complex concentrates (pd-APCC). With the efficacy of these agents now well established, a number of health economic studies have been conducted to compare their cost-effectiveness for the on-demand treatment of bleeding episodes in hemophiliacs with inhibitors. In a cost-utility analysis, which assesses the effects of treatment on quality of life (QoL) and quantity of life, the incremental cost per quality-adjusted life-year (QALY) gained (US $44,834) indicated that rFVIIa was cost-effective. Similarly, eight of 11 other economic modelling evaluations found that rFVIIa was more cost-effective than pd-APCC in the on-demand treatment of bleeding episodes. The findings of Escobar indicated that treating patients with hemophilia promptly and with the most effective therapy available may result in cost savings.

Although hemophilia is an expensive disorder, no studies have estimated health care costs for Americans with hemophilia enrolled in Medicaid as distinct from those with employer-sponsored insurance (ESI) (GUH). The study of Guh et al. [25] provided information on health care utilization and expenditures for publicly insured people with haemophilia in the United States in comparison with people with haemophilia who have ESI. Data from the MarketScan Medicaid Multi-State, Commercial and Medicare Supplemental databases were used for the period 2004–2008 to identify cases of hemophilia and to estimate medical expenditures during 2008. A total of 511 Medicaid-enrolled males with hemophilia were identified, 435 of whom were enrolled in months during 2008. Most people with Medicaid for at least 11 hemophilia qualified for Medicaid based on “disability”. Average Medicaid expenditures in 2008 were $142,987 (median, $46,737), similar to findings for people with ESI. Average costs for males with hemophilia A and an inhibitor were 3.6 times higher than those for individuals without an inhibitor. Average costs for 56 adult Medicaid enrollees with HCV or HIV infection were not statistically different from those for adults without the infection, but median costs were 1.6 times higher for those treated for blood-borne infections. Hemophilia treatment can lead to high costs for payers. Further research is needed to understand the effects of public health insurance on hemophilia care and expenditures, to evaluate treatment strategies and to implement strategies that may improve outcomes and reduce costs of care.

## 4. Conclusions

Hematologic prophylactic treatment from ages 2 to 18 years could avoid the development of hemophilic arthropathy if the concentration of the patient's deficient factor is prevented from falling below 1% of normal [26–28]. Early treatment is of paramount importance because the immature skeleton is very sensitive to the complications of hemophilia; severe structural deficiencies may develop quickly. Major hemarthrosis and chronic hemophilic synovitis should be treated aggressively to prevent hemophilic arthropathy [29–31].
